# Intubation in Eosinophilic Lung Disease: Predictors, Outcomes, and Characteristics from a National Inpatient Sample Analysis

**DOI:** 10.3390/medicina61040556

**Published:** 2025-03-21

**Authors:** Michel Al Achkar, Nadim Zaidan, Chloe Lahoud, Zaineb Zubair, Jessica Schwartz, Erica Abidor, Chris Kaspar, Halim El Hage

**Affiliations:** 1 Department of Medicine, Northwell Health, Staten Island University Hospital, New York, NY 10305, USA; nzaidan@northwell.edu (N.Z.); chloelahoud@gmail.com (C.L.); zzubair@northwell.edu (Z.Z.); jschwartz15@northwell.edu (J.S.); eabidor@northwell.edu (E.A.); ckaspar@northwell.edu (C.K.); 2 Department of Pulmonary Medicine, Northwell Health, Staten Island University Hospital, New York, NY 10305, USA; helhage@northwell.edu

**Keywords:** eosinophilic pneumonia, mortality, intubation, acute kidney injury

## Abstract

*Background and Objectives*: Eosinophilic lung diseases (ELD) encompass disorders with an abnormally high number of polymorphonuclear eosinophils in the lungs. Presentation severity can range from low-grade fever and cough to life-threatening acute respiratory distress syndrome (ARDS). Due to the rarity of these conditions, no large sample studies have been performed to assess the characteristics of patients with pulmonary eosinophilia. *Materials and Methods*: Patients admitted with a diagnosis of pulmonary eosinophilia between the years 2016 and 2020 were extracted from the largest inpatient US database, the Nationwide Inpatient Sample (NIS). Patients under the age of eighteen and those with diabetic ketoacidosis were excluded. Baseline demographic characteristics and medical comorbidities were evaluated for individuals admitted with pulmonary eosinophilia depending on intubation requirement. The primary outcomes included in-hospital mortality, intubation, and length of stay (LOS). *Results*: 3784 records were extracted, among which 384 patients required intubation. Patients who required intubation had higher rates of in-hospital mortality (23.9% vs. 1.2% *p* < 0.0001%) and a significantly more prolonged hospital stay (19 days vs. 6 days *p* < 0.001) compared to patients who did not need intubation. Factors associated with mortality in the intubated group included increasing age (OR: 1.022, 95% CI 1.002–1.042), duration of intubation superior to 96 h (OR: 2.705, 95% CI 1.235–5.927), and AKI (OR: 2.964, 95% CI 1.637–5.366). *Conclusions*: Our findings suggest that ELD patients requiring intubation experience significantly higher rates of in-hospital mortality, acute kidney injury, deep venous thrombosis, and ARDS.

## 1. Introduction

Eosinophilic lung diseases are a group of disorders characterized by the abnormal accumulation of polymorphonuclear eosinophils (PNEs) in the lung tissues, airways, or both. PNEs are primarily involved in the immune response against parasitic infections; they also play a role in asthma and allergic reactions [1]. ELD can manifest as either acute or chronic conditions and two major types of eosinophilic pneumonia dominate this category: acute eosinophilic pneumonia (AEP) and chronic eosinophilic pneumonia (CEP) [2]. Both conditions are interstitial lung diseases marked by eosinophilic infiltration, though they differ significantly in clinical presentation and progression [2].

AEP typically develops rapidly, often within days, and is characterized by fever, cough, and dyspnea. Triggers may include environmental exposures, such as smoking, inhaled antigens, or medications [3]. Severe cases may mimic acute respiratory distress syndrome (ARDS) [4]. The data on the incidence of AEP is scarce but small studies report an incidence of 11 per 100,000 person/year [5]. In contrast, CEP develops insidiously over weeks to months and is more common in individuals with a history of asthma or atopy. Symptoms include progressive shortness of breath and weight loss [6]. It also has a lower reported incidence than AEP, with a small study in Iceland estimating the incidence of CEP at 0.23–0.54 per 100,000 person/year [7].

Eosinophilic lung diseases also encompass allergic bronchopulmonary aspergillosis (ABPA) [8], commonly associated with fungal hypersensitivity, and systemic disorders such as eosinophilic granulomatosis with polyangiitis (EGPA) [9]. Infectious etiologies, particularly parasitic infections, and drug-induced pulmonary eosinophilia must also be considered during differential diagnosis [2].

Alveolar cell injury in eosinophilic pneumonia triggers an inflammatory cascade, leading to the secretion of IL-33, which promotes the recruitment and activation of PNEs in the lungs [10]. The recruitment of T-helper 2 (TH2) cells by the inflammatory cascade leads to the production of IL-5, which further promotes the influx of eosinophils into the lungs [3].

Eosinophilic lung diseases are rare but potentially life-threatening if misdiagnosed or untreated. Corticosteroids constitute the cornerstone of eosinophilic lung disease treatment; although, no specific treatment regimens have been established [11]. One special challenge in treating patients with eosinophilic lung disease is the ability to stratify patients based on the severity of their disease. Individuals with eosinophilic lung disease developing ARDS and requiring intubation may be at an increased risk of complications [12].

In this study, we aim to evaluate the factors associated with intubation and mortality in ELD. We also seek to obtain a more thorough understanding about the intricate interplay between multiple comorbidities—such as asthma, chronic obstructive pulmonary disease, heart rhythm abnormalities, and acute and chronic kidney disease—and ELD.

## 2. Materials and Methods

We conducted a cross-sectional analysis using the Nationwide Inpatient Sample (NIS) for the years 2016 to 2020. Containing information on over seven million hospital visits per year, the NIS is the largest publicly accessible inpatient care database in the United States. The size of NIS database makes it an invaluable tool for researching conditions with low prevalence such as eosinophilic lung diseases [13]. Since the data included in the database is de-identified, this study did not require institutional review board approval.

The International Classification of Diseases, 10th version (ICD-10) codes were used to identify all records containing a diagnosis of pulmonary eosinophilia or eosinophilic pneumonia. Age < 18 years and diabetic ketoacidosis were used for exclusion. Demographic information such as age, sex, and race in addition to different comorbidities were extracted for patients that met inclusion criteria. ICD-10-PCS Code Description and Clinical Classifications Software Refined (CCSR) were used for identifying occurrence of intubation and mechanical ventilation. The diagnoses and procedures codes used for extraction are summarized in Table A1 in Appendix A.

The primary outcomes included in-hospital mortality and length of stay (LOS). Secondary outcomes were acute kidney injury (AKI), pulmonary embolism (PE), deep venous thrombosis (DVT), and acute respiratory distress syndrome (ARDS).

Analysis using the complex sample feature of SAS (Survey Procedures Package) allowed us to account for 3 factors specific to the NIS database: discharge weights (DISCWT), as well as hospital clustering (HOSP_NIS) and stratification (NIS_STRATUM).

Age and LOS are presented as means with a standard error of mean; both groups were compared using the Wilcoxon test. Categorical variables are presented as percentages with standard errors and comparisons were conducted through second order Rao–Scott chi-square tests.

Multivariate binary logistic regressions were computed to evaluate the strength and significance of the relationship between the covariates of interests and two outcomes: intubation and mortality. The covariates that we accounted for included demographic variables such as age, sex, and race, as well as multiple comorbidities such as diabetes, hypertension, obesity, chronic obstructive pulmonary disease (COPD), cigarette use, hypothyroidism, chronic kidney disease (CKD), end stage kidney disease (ESKD), cannabis use, chronic non-steroidal anti-inflammatory drug use, human immunodeficiency virus (HIV) infection, asthma, vape use, atrial fibrillation (AFIB), coronary artery disease, and obstructive sleep apnea (OSA). Regression results are presented as odds ratio (OR) estimates with 95% confidence intervals. We report statistical parameters relevant to the regression models, specifically the c-statistic, which evaluates the discriminatory ability of the model. Results for the likelihood ratio tests are additionally reported to show regression model fitness.

All statistical analyses were conducted using SAS Enterprise Software, version 9.4 (SAS Institute Inc., Cary, NC, USA). A two-sided *p*-value of less than 0.05 was deemed statistically significant.

## 3. Results

### 3.1. Clinical Characteristics of Patients with ELD

Clinical characteristics of the cohort are summarized in Table 1. A total of 3784 patients were included, of whom 384 required intubations. Significant differences were observed in the age and gender between the 2 cohorts, with intubated group being slightly younger, with a mean age of 58.9 years (*p*: 0.0188), and having an almost equal proportion of males and females (female proportion 49.7%; *p*: 0.0243). A lower prevalence of hypertension, COPD, hypothyroidism, and OSA was identified in the intubated cohort (*p* < 0.05). The prevalence of AFIB was higher in the intubated group (23% vs. 18%; *p*: 0.022).

### 3.2. Clinical Outcomes According to Intubation Status in ELD

The outcome occurrence based on intubation status is presented in Table 2. Patients with eosinophilic lung disease who required intubation had a significantly more elevated mortality (23.9% vs. 1.2%; *p* < 0.001) and a significantly more prolonged LOS (19 days vs. 6 days; *p* < 0.001) compared to patients who did not need intubation. Patients who required intubation had a higher rate of AKI (51.3% vs. 16.2%; *p* < 0.001), DVT (3.9% vs. 1%; *p* < 0.0001), and ARDS (14.3% vs. 0.5%; *p* < 0.001). The rate of pulmonary embolism (PE) was slightly more elevated in the intubated cohort, but it did not reach statistical significance (3.3% vs. 2%; *p*: 0.1747).

Using the PWR package in R (2023.12.1+402) and running the intended power analysis for multivariate binary logistic regression for the outcome of intubation compared to no intubation, our study, including a population of 3784, a significance level of 0.05, and using 19 predictors, was adequately powered (0.98) to detect an effect size of 0.01.

### 3.3. Factors Associated with Mortality in Patients with ELD

We assessed patients that were admitted with ELD regardless of intubation status for factors associated with mortality. These results are presented in Table 3. These factors include increasing age (OR: 1.027; 95% CI 1.011–1.044), AFIB (OR: 1.873; 95% CI 1.162–3.019), intubation (OR: 18.603; 95% CI 11.853–29.195), and AKI (OR: 3.662; 95% CI 2.296–5.841).

### 3.4. Covariates Associated with Intubation in Patients with ELD

We looked for covariates associated with intubation in patients admitted with ELD. Results of the multivariate logistic regression are presented in Table 4. These covariates include: Age (OR: 0.984; 95% CI 0.976–0.991),black race (OR: 0.661; 95% CI 0.450–0.969), hypothyroidism (OR: 0.565; 95% CI 0.372–0.856), AFIB (OR: 1.4; 95% CI 1.015–1.951), OSA (OR: 0.531 95% CI; 0.358–0.787), AKI (OR: 6.418; 95% CI 4.882–8.437), ARDS (OR: 25.974; 95% CI 13.114–51.446), DVT (OR: 2.549; 95% CI 1.016–6.396).

### 3.5. Factors Associated with Mortality in Intubated Patients with Eosinophilic Lung Disease

Focusing solely on the intubated group to identify which factors were associated with mortality, relevant covariates included increasing age (OR: 1.022, 95% CI 1.002–1.042),black race (OR: 0.327, 95% CI 0.108–0.992), duration of intubation of more than 96 h (OR: 2.705, 95% CI 1.235–5.927), and AKI (OR: 2.964, 95% CI 1.637–5.366). Results for this regression analysis are presented in Table 5.

### 3.6. Variables Associated with ARDS in Patients with ELD

Finally, we evaluated patients that were admitted with ELD regardless of intubation status for factors that were associated with ARDS (Table 6). We found that COPD was associated with a lower odds ratio of ARDS (OR: 0.216, 95% CI 0.053–0.890) and intubation and ARDS had a strong positive association (OR: 31.2, 95% CI 17.5–58.168).

## 4. Discussion

Although ELD is a relatively uncommon condition, our analysis of the NIS database identified a significant number of patients admitted due to this diagnosis. Furthermore, our findings are generalizable since they are based on a database that contains indicators from hospitals across the United States, which encompass a diverse patient population. It is critical to understand which factors are associated with intubation and increased mortality in patients with ELD. While age was found to be inversely correlated with risk of intubation in patients with ELD, our data suggest that patients with ELD who required intubation had a higher prevalence of AKI, AFIB, and DVTs. Our study also found that patients with eosinophilic lung disease and coexisting COPD were observed to have a lower risk of developing ARDS during hospitalization. AKI, AFIB, and longer duration of intubation were found to be associated with an increased mortality.

AKI was associated with an increased odds ratio of intubation. The occurrence of AKI can lead to a state of fluid overload which could eventually lead to worsening of respiratory status and ventilation mechanics eventually leading to intubation [14,15]. The clinical characteristics of patients in our study revealed that AKI was more prevalent among patients with eosinophilic lung disease who required intubation, compared to those who did not. Hypoxia resulting from severe eosinophilic lung disease requiring intubation may have significantly contributed to the development of AKI in this cohort [16]. Peri-intubation adverse events such as hypotension or cardiovascular collapse could also be a contributing factor for the increased prevalence of AKI in this group. Our findings align with the existing literature, including a study by McNicholas et al., which demonstrated that AKI could develop within 24 h after intubation in COVID-19 patients experiencing respiratory failure [17].

AFIB was also found to be more prevalent in intubated patients with ELD. Patients with ELD and AFIB can be at an increased risk of intubation as AFIB can be a sign of cardiovascular instability in critically ill patients [18]. AFIB can also result from infiltration of the heart muscle by PNE, with multi-organ involvement serving as an indicator of severe disease [19]. Patients with severe eosinophilic pneumonia are usually treated with higher doses of intravenous steroids [20]. In the published literature, higher doses of intravenous steroids were found to be associated with new onset AFIB in patients with asthma [21]. Van Der Hooft et al. also report that asthmatic patients treated with doses of steroids had an increased risk of AFIB [22]. In addition, the higher incidence of arrhythmogenic precipitants in patients who are intubated or hospitalized to the intensive care unit may also be the cause of the elevated prevalence of AFIB in the intubated cohort [18].

Although there was an observed increase in the incidence of PE, this difference was not statistically significant, as the *p*-value exceeded the threshold for significance. Ibrahim et al. found that intubation was associated with an increased risk of DVT despite being on DVT prophylaxis [23]. Patients admitted to intensive care units with ARDS caused by COVID-19 infection or pneumonia were particularly susceptible to DVT [24,25]. ARDS, which was more prevalent in our intubated cohort, further amplifies the risk of DVT by increasing inflammatory markers and disrupting the coagulation cascade, fostering a pro-thrombotic state [26].

Among patients admitted with ELD, patients who had COPD were found to have a lower odds ratio of developing ARDS. COPD has traditionally been associated with an increased risk of bacterial pneumonia, one of the most common causes of ARDS [27,28]. Patients with chronic COPD are typically treated with steroid-containing inhalers. Both COPD and ELD can be managed with oral steroids [10,29], potentially explaining our intriguing findings. Eosinophils vanish relatively quickly following steroid treatment [2], and administration of steroids for patients with both COPD and ELD could help prevent progression to ARDS. The data on the potential benefits of steroids for ARDS patients is debatable; these benefits are most likely to be observed during the acute stages of ARDS [30]. Regarding inhaled steroids, pre-admission use of inhaled steroids was associated with a decreased risk of developing ARDS and acute lung injury in a study by Ortiz-Diaz et al. [31]. Another study showed that inhaled budesonide was associated with improvement in ventilation parameters in individuals with ARDS [32].

In our analysis, it was shown that hypothyroidism was associated with a lower odds ratio of intubation. Levothyroxine, a commonly used thyroid hormone replacement has been proven to have an anti-inflammatory effect which might explain the negative association with intubation in patients with hypothyroidism [33]. OSA was also found to be associated with a lower odds ratio of intubation, although classically OSA and obesity convey a higher risk of intubation in other pulmonary diseases such as COVID-19 [34,35]. Our contradictory results may be explained by the fact that OSA provokes a Th1-mediated inflammatory response in the airways, characterized by neutrophil infiltration in the lungs, as well as an elevated neutrophil count observed in the blood [36]. This might skew the inflammatory response away from the Th2 and eosinophilic pathway. In addition, patients with OSA are usually treated with continuous positive airway pressure or bi-level positive airway pressure ventilation to overcome the airway obstruction [37].

Older age was found to be associated with mortality. This association might be attributed to the fact that elderly ELD patients often have more comorbidities and are more susceptible to complications and are at risk of increased mortality in the intensive care setting [38]. A study conducted in Japan found that elderly patients admitted with eosinophilic pneumonia had a prolonged duration of corticosteroid use and a delay in the initiation of steroids after admission [39] which may increase complications and mortality in this specific population. Age was found to be inversely correlated with the need for intubation. Acute eosinophilic pneumonia generally occurs in younger individuals and is characterized by a more severe and rapidly progressive course compared to chronic eosinophilic pneumonia [10]. Due to its acute and aggressive nature, patients with acute eosinophilic pneumonia are more likely to need intubation [3].

In our study, a higher proportion of patients in the intubated group were diagnosed with ARDS. A strong association between ARDS and the need for intubation was seen in our analysis, this is likely due to the severe respiratory compromise seen in ARDS, which often necessitate mechanical ventilation to maintain oxygenation [40].

The length of stay for the intubated cohort was 19 days, significantly longer than the 6-day stay observed in patients who did not require intubation. Individuals who required intubation due to ELD in our analysis had an increased incidence of AKI and ARDS, which might require prolonged hospitalization for prolonged treatment. Another factor that may contribute to the increased length of stay is the prolonged duration of treatment for ELD patients requiring intubation. Carbone et al. recommended that severe eosinophilic pneumonia should be treated with intravenous methylprednisolone [2], which can significantly prolong hospital stay.

Intubation for more than 96 h was associated with an increased odds ratio of mortality. A prolonged duration of intubation is associated with an increased risk of developing complications such as ventilator-acquired pneumonia, with longer durations of intubation associated with nosocomial infections with multi-drug resistant organisms [41]. The occurrence of pneumothorax in intubated patients with ARDS was found to be associated with increased mortality, with the risk of developing pneumothorax rising with longer durations of intubation [42].

Significantly higher mortality was witnessed in individuals with ELD requiring intubation. This observation may be explained by multiple factors. Severe eosinophilic pneumonia requiring intubation can be complicated by concurrent pneumonias and can exacerbate underlying conditions such as heart failure or COPD, conveying a high risk or mortality. Intubation has also been associated with higher mortality rates in different pulmonary pathologies such as pneumonia [43] and asthma [44]. The increased mortality in our study can also be explained by the higher prevalence of AFIB and DVT seen in patients requiring intubation. AFIB and DVT treatment require systemic anticoagulation, placing patients at an inherent risk of major bleeding and, therefore, mortality [45,46]. AKI, which was more prevalent in patients requiring intubation, was also identified as an independent risk factor for mortality in intubated ARDS patients in a study by Fadel et al. [47].

Patients with ELD requiring intubation face significantly higher mortality and complications like acute kidney injury, deep vein thrombosis, and ARDS, necessitating aggressive monitoring and proactive treatment. Risk stratification based on factors like older age, atrial fibrillation, and AKI can guide earlier, more aggressive interventions to potentially prevent disease progression and intubation. Minimizing intubation duration is crucial, as prolonged mechanical ventilation increases mortality risk. Further research is essential to optimize ELD management strategies, especially for severe cases requiring intubation, and to investigate the interplay of ELD with comorbidities like COPD.

Various limitations to our study should be taken into account. The NIS database may have included coding errors since it relies on ICD-10 coding. Patients with acute, chronic, and unspecified pulmonary eosinophilia were pooled in our analysis. This grouping might weaken differences in outcomes among subgroups of pulmonary eosinophilia. While dividing the included patients into groups according to the cause of pulmonary eosinophilia might have provided more detailed insights, the small sample size made this impracticable and would have reduced the statistical power of our results.

Treatment details, including the use of steroids and antimicrobials, as well as occupational and exposure history, could not be accounted for as they are not included in the NIS database. The timing of events, like intubation or the onset of ARDS, during the hospital course could not be accounted for either. Another limitation resides in the lack of laboratory values such as white blood cell count, polymorphonuclear eosinophil count, and inflammatory markers. Since the NIS relies on data retrieved from hospital admission, we could not account for mild cases of eosinophilic pneumonia that may have been treated without the need of being admitted or that simply went undiagnosed. In conclusion, these limitations show that more extensive and prospective research is required to completely comprehend the challenges in managing patients with ELD.

## 5. Conclusions

In conclusion, our analysis reveals that intubated individuals with ELD have a much higher rate of adverse outcomes including DVT, AKI, and AFIB, and experience higher mortality than individuals with eosinophilic lung disease who do not require intubation; in addition, patients with COPD were less likely to develop ARDS from ELD. By identifying the predictors of mortality and ARDS, we uncover characteristics that can stratify individuals admitted with ELD based on disease severity to better decide on treatment strategies. Early management of those complications and efforts to shorten the intubation period may be linked to improved survival. After identifying those risk factors, clinicians may decide to take a more aggressive approach in ELD treatment. This study highlights the challenges and complexity of managing ELD in patients requiring intubation. Our study also stresses the need to comprehend the mechanisms by which several risk factors exacerbate the disease’s severity. Multicenter prospective trials are needed to better characterize risk factors associated with severe eosinophilic lung disease to guide management, especially given the absence of treatment guidelines.

## Figures and Tables

**Table 1:** Clinical characteristics of individuals admitted with eosinophilic lung disease (ELD).

Variables	Eld Not Requiring Intubation	ELD Requiring Intubation	*p*-Value
*n*	3400	384	
Age (mean, SE of M)	61.2 (0.316)	58.9 (0.850)	0.0188 *
Female sex (%)	1898 (55.8%)	191 (49.7%)	0.0243 *
Race (%)			0.15
White	2333 (68.6%)	258 (67.1%)	
Black	464 (13.6%)	42 (10.9%)	
Hispanic	278 (8.1%)	42 (10.9%)	
Other	222 (6.5%)	26 (6.7%)	
Diabetes Mellitus (%)	921 (27%)	111 (28.9%)	0.4621
Hypertension (%)	1119 (32.9%)	99 (25.7%)	0.0033 *
Obesity (%)	420 (12.3%)	44 (11.4%)	0.6109
Chronic obstructive pulmonary disease (%)	476 (14%)	36 (9.3%)	0.0036
Cigarette use (%)	413 (12.1%)	47 (12.2%)	0.9581
Hypothyroidism (%)	488 (14.3%)	30 (7.8%)	<0.0001 *
Chronic kidney disease (%)	410 (12%)	52 (13.5%)	0.4177
End stage renal disease (%)	68 (2%)	11 (2.8%)	0.3256
Cannabis use (%)	74 (2.1%)	5 (1.3%)	0.160
Chronic non-steroidal anti-inflammatory drug use (%)	30 (0.8%)	4 (1%)	0.7701
HIV (%)	25 (0.7%)	2 (0.5%)	0.5878
Asthma (%)	214 (6.2%)	17 (4.4%)	0.0959
Vape use (%)	13 (0.3%)	1 (0.2%)	0.6676
Atrial fibrillation (%)	613 (18%)	89 (23%)	0.0220 *
Coronary artery disease (%)	713 (20%)	67 (17.4%)	0.0828
Obstructive sleep apnea (%)	532 (15.6%)	37 (9.6%)	0.0002 *

The NIS databases were initially combined for the years 2016 to 2020. Hospital records were classified according to whether patients admitted with eosinophilic lung disease underwent intubation. Age is reported as mean with the standard error of the mean, and the *p*-value is obtained from the Wilcoxon test. The remaining covariates are presented as percentages with the standard error of the percentage. *p*-values are from second order chi-square (Rao–Scott chi-square test). *: denotes statistical significance.

**Table 2:** Comparison of clinical outcomes in patients admitted with ELD depending on intubation status.

ELD	Not Intubated	Intubated	*p* Value
*n*	3400	384	
In-hospital mortality (%)	44 (1.2%)	92 (23.9%)	<0.0001 *
ARDS (%)	18 (0.5%)	55 (14.3%)	<0.0001 *
Deep venous thrombosis (%)	36 (1%)	15 (3.9%)	0.0048 *
Acute kidney injury (%)	552 (16.2%)	197 (51.3%)	<0.0001 *
Pulmonary embolism (%)	71 (2%)	13 (3.3%)	0.1747
Length of stay (mean, SE of M)	6.3 (0.128)	19 (0.883)	<0.0001 *

Originally, the NIS databases were combined for the years 2016–2020. Hospital records were categorized based on whether or not intubation was performed on patients who were admitted with eosinophilic lung disease. The length of stay is reported as the mean with the standard error of the mean, and the *p*-value is obtained from the Wilcoxon test. For the remaining outcomes the data are presented with counts and percentages, and *p*-values are from second order chi-square (Rao–Scott chi-square test). *: denotes statistical significance.

**Table 3:** Multivariate binary logistic regression for the outcome of mortality among all hospitalized ELD patients.

Covariate		Estimate OR	Lower Interval	Upper Interval
Age		1.027	1.011	1.044 *
Sex (ref = male)		1.074	0.715	1.614
Race (ref = white)				
	Black	0.732	0.342	1.567
	Hispanic	1.129	0.565	2.256
	Asian	0.594	0.181	1.95
	Native American	<0.001	<0.001	<0.001
	Other	3.126	1.172	8.339 *
Diabetes Mellitus		0.946	0.591	1.513
Hypertension		0.717	0.436	1.18
Obesity		1.200	0.637	2.261
Chronic obstructive pulmonary disease		1.030	0.547	1.938
Cigarette use		0.550	0.214	1.413
hypothyroidism		0.760	0.393	1.47
Chronic kidney disease		0.650	0.354	1.193
Asthma		0.374	0.095	1.472
Atrial fibrillation		1.873	1.162	3.019 *
Coronary artery disease		1.490	0.913	2.431
Intubation status		18.603	11.853	29.195 *
Obstructive sleep apnea		0.605	0.279	1.314
ESRD		0.727	0.218	2.429
Acute kidney injury		3.662	2.296	5.841 *
ARDS		0.606	0.27	1.361
Deep venous thrombosis		2.630	0.797	8.680
Pulmonary embolism		0.919	0.269	3.141

Odds ratios for multiple covariate factors in hospitalized patients with ELD for the outcome of inpatient mortality, irrespective of intubation status. These ORs with 95% confidence interval resulted from multivariate binary logistic regression accounting from all the covariates mentioned in the table. C-statistic = 0.913. Likelihood ratio test: F Value 16.56 (Pr > F < 0.0001; second order Rao-Scott design correction 0.0271 applied to the likelihood ratio test). *: denotes statistical significance.

**Table 4:** Multivariate binary logistic regression for the outcome of intubation among all hospitalized ELD patients.

Covariate		Estimate OR	Lower Interval	Upper Interval
Age		0.984	0.976	0.991 *
Sex (ref = male)		1.007	0.785	1.291
Race (ref = white)				
	Black	0.661	0.450	0.969 *
	Hispanic	1.253	0.848	1.85
	Asian	0.461	0.179	1.186
	Native American	0.869	0.213	3.545
	Other	0.996	0.540	1.839
Diabetes Mellitus		0.995	0.739	1.339
Hypertension		0.939	0.707	1.247
Obesity		1.162	0.798	1.692
Chronic obstructive pulmonary disease		0.854	0.582	1.253
Cigarette use		0.797	0.539	1.176
Hypothyroidism		0.565	0.372	0.856 *
Asthma		0.766	0.431	1.361
Atrial fibrillation		1.407	1.015	1.951 *
Coronary artery disease		0.808	0.577	1.130
Obstructive sleep apnea		0.531	0.358	0.787 *
ESRD		1.358	0.628	2.937
Acute kidney injury		6.418	4.882	8.437 *
ARDS		25.974	13.114	51.446 *
Deep venous thrombosis		2.549	1.016	6.395 *
Pulmonary embolism		1.060	0.491	2.289

Odds ratios for multiple covariate factors in hospitalized patients with ELD with regard to intubation. These ORs with 95% confidence interval resulted from multivariate binary logistic regression accounting from all the covariates mentioned in the table. C-statistic = 0.784. Likelihood ratio test: F Value 18.93 (Pr > F < 0.0001; second order Rao-Scott design correction 0.0107 applied to the likelihood ratio test). *: denotes statistical significance.

**Table 5:** Multivariate binary logistic regression for the outcome of mortality among intubated ELD patients.

Covariate		Estimate OR	Lower Interval	Upper Interval
Age		1.022	1.002	1.042
Sex (ref = male)		1.234	0.706	2.158
Race (ref = white)				
	Black	0.327	0.108	0.992 *
	Hispanic	0.851	0.345	2.096
	Asian	0.504	0.094	2.706
	Native American	<0.001	<0.001	<0.001
	Other	2.041	0.526	7.917
Diabetes Mellitus		0.812	0.441	1.495
Hypertension		0.836	0.452	1.546
Obesity		1.268	0.524	3.073
Chronic obstructive pulmonary disease		0.826	0.335	2.039
Cigarette use		0.343	0.107	1.101
Hypothyroidism		1.140	0.423	3.070
Chronic kidney disease		0.615	0.268	1.412
Atrial fibrillation		1.458	0.78	2.725
Coronary artery disease		1.663	0.771	3.585
Duration of intubation	(ref: <24 h)			
	24 to 96 h	1.438	0.646	3.199
	>96 h	2.705	1.235	5.927 *
Acute kidney injury		2.964	1.637	5.366 *
ARDS		0.562	0.261	1.208
Deep venous thrombosis		0.553	0.15	2.036
Pulmonary embolism		0.658	0.142	3.042

Odds ratios for multiple covariate factors in hospitalized patients with ELD with regard to mortality among intubated individuals. These ORs with 95% confidence interval resulted from multivariate binary logistic regression accounting from all the covariates mentioned in the table. C-statistic = 0.776. Likelihood ratio test: F Value 3.33 (Pr > F < 0.0001; second order Rao-Scott design correction 0.0992 applied to the likelihood ratio test). *: denotes statistical significance.

**Table 6:** Multivariate binary logistic regression for the outcome of developing ARDS among patients admitted with ELD.

Covariate		Estimate OR	Lower Interval	Upper Interval
Age		1	0.983	1.018
Sex (ref = male)		0.825	0.507	1.344
Race (ref = white)				
	Black	0.825	0.507	1.344
	Hispanic	1.347	0.645	2.814
	Asian	2.656	0.797	8.848
	Native American	1.567	0.162	15.120
	Other	1.286	0.400	4.131
Diabetes Mellitus		1.125	0.648	1.953
Hypertension		0.652	0.342	1.243
Obesity		0.516	0.185	1.440
Chronic obstructive pulmonary disease		0.215	0.053	0.875 *
Cigarette use		1.631	0.736	3.616
Hypothyroidism		1.357	0.595	3.092
Asthma		1.342	0.478	3.769
Atrial fibrillation		1.342	0.713	2.525
Coronary artery disease		0.647	0.308	1.356
Intubation status		31.942	17.518	58.242 *

Odds ratios for multiple covariate factors in hospitalized patients with ELD with regard to developing ARDS. These ORs with a 95% confidence interval resulted from multivariate binary logistic regression accounting from all the covariates mentioned in the table. C-statistic = 0.886. Likelihood ratio test: F Value 11.12 (Pr > F < 0.0001; second order Rao-Scott design correction 0.0107 applied to the likelihood ratio test). *: denotes statistical significance.

## Data Availability

The original contributions presented in this study are included in the article. Further inquiries can be directed to the corresponding author.

## Abbreviations

The following abbreviations are used in this manuscript:

AFIB	Atrial fibrillation
AKI	Acute kidney injury
ELD	Eosinophilic lung disease
ARDS	Acute respiratory distress syndrome
AEP	Acute eosinophilic pneumonia
CEP	Chronic eosinophilic pneumonia
NIS	Nationwide Inpatient Sample
LOS	Length of stay
PNE	Polymorphonuclear eosinophils
ICD	10 The International Classification of Diseases, 10th version
PE	Pulmonary embolism
DVT	Deep venous thrombosis
HIV	human immunodeficiency virus
AFIB	Atrial fibrillation
OSA	Obstructive sleep apnea

## Appendix A

**Table A1:** ICD-10 codes used in our analysis.

J82 J8281 J8282 J8289	Eosinophilic lung disease
E0821, E0822, E0840, E0842, E0843, E0851, E0852, E08621, E08649, E0865, E0869, E088, E089, E1010, E1011, E1021, E1022, E1029, E10311, E10319, E103213, E103219, E103291, E103292, E103293, E103299, E103313, E103393, E103399, E103413, E103493, E103499, E103511, E103512, E103513, E103519, E103591, E103592, E103593, E103599, E1036, E1039, E1040, E1041, E1042, E1043, E1044, E1049, E1051, E1052, E1059, E10610, E10618, E10620, E10621, E10622, E10628, E10638, E10641, E10649, E1065, E1069, E108, E109, E1100, E1101, E1110, E1111, E1121, E1122, E1129, E11311, E11319, E113211, E113212, E113213, E113219, E113291, E113292, E113293, E113299, E113311, E113312, E113313, E113319, E113391, E113392, E113393, E113399, E113411, E113412, E113413, E113419, E113491, E113492, E113493, E113499, E113511, E113512, E113513, E113519, E113522, E113523, E113533, E113551, E113552, E113553, E113591, E113592, E113593, E113599, E1136, E1137X9, E1139, E1140, E1141, E1142, E1143, E1144, E1149, E1151, E1152, E1159, E11610, E11618, E11620, E11621, E11622, E11628, E11630, E11638, E11641, E11649, E1165, E1169, E118, E119, E1300, E1301, E1310, E1311, E1321, E1322, E13319, E1336, E1339, E1340, E1342, E1343, E1351, E1352, E1359, E13610, E13621, E13622, E13628, E13649, E1365, E1369, E138, E139	Diabetes Mellitus
I10, I150, I151, I152, I158, I159	Hypertension
G4733	Obstructive sleep apnea (adult) (pediatric)
E6609, E661, E662, E663, E668, E669	Obesity
J410, J42, J439, J449	Chronic obstructive pulmonary disease
Z720, F17200, F17209, F17210, F17218, F17219	Smoking
E032, E039, E038	Hypothyroidism
I82401, I82402, I82403, I82409, I82411, I82412, I82413, I82419, I82421, I82422, I82423, I82429, I82431, I82432, I82433, I82439, I82491, I82492, I82493, I82499, I824Y1, I824Y2, I824Y3, I824Y9	Acute deep venous thrombosis
N170, N178, N179	Acute kidney injury
N181, N182, N183, N1830, N1831, N1832, N184, N185	Chronic kidney disease
N186	End stage kidney disease
J80	Acute respiratory distress syndrome
Z791	Long term (current) use of non-steroidal anti-inflammatory drugs
Z21, B20	Human immunodeficiency virus infection
F1210, F12129, F12120, F1220, F1290	Cannabis use
J4520, J4530, J4540, J4550, J45909	Asthma
U070	Vape use
I480, I481, I4811, I4819, I482, I4820, I4821, I483, I484, I4891, I4892	Atrial fibrillation
I2510, I25110, I25111, I25118, I25119, I252, I255, I256, I2582, I2583, I2584	Asthma
0BH17EZ	Intubation
I2609, I2692, I2693, I2694, I2699	Pulmonary embolism
